# The Translational Medicine Regarding Ozone in Saline Solutions. Comment on Armeli et al. Ozone Saline Solution Polarizes Microglial Cells Towards an Anti-Inflammatory Phenotype. *Molecules* 2025, *30*, 3932

**DOI:** 10.3390/molecules31071187

**Published:** 2026-04-03

**Authors:** Marianno Franzini, Luigi Valdenassi, Salvatore Chirumbolo

**Affiliations:** 1 Italian Scientific Society of Oxygen-Ozone Therapy (SIOOT), 24020 Bergamo, Italy; marianno.franzini@gmail.com (M.F.); luigi.valdenassi@unipv.it (L.V.); 2 Department of Engineering for Innovation Medicine, University of Verona, 37134 Verona, Italy

**Keywords:** ozone, ozone in saline solution, comment, therapy, bias

## Abstract

This commentary critically evaluates the translational relevance of a recent study investigating the effects of ozonated saline solution (O_3_SS) on microglial and endothelial cell models. While the original research proposes potential antioxidant and anti-inflammatory benefits of low-dose ozone exposure, we identify significant methodological and conceptual flaws that undermine its conclusions. Key concerns include the unjustified assumption that ozone behaves similarly in microwell cultures and clinical infusion settings, despite known physicochemical differences affecting ozone stability and reactivity. The use of immortalized BV2 and HUVEC cells, which lack the complexity of in vivo systems, further limits the study’s applicability. The absence of accurate ozone quantification, proper controls, protein-level validation, and kinetic modeling exacerbates these weaknesses. Our analysis also demonstrates, through differential equation modeling, that ozone rapidly decays in saline solutions, making systemic delivery via infusion chemically implausible as a therapeutic approach. Moreover, the extrapolation of in vitro gene expression data to systemic therapeutic claims lacks scientific justification. We conclude that while the observed cellular responses in vitro are of academic interest, they do not support the efficacy or safety of O_3_SS in clinical settings. A more rigorous approach is necessary to substantiate the biomedical potential of ozonated solutions.

## 1. Introduction

A recent article by Armeli et al. presents itself as a preclinical investigation into the putative antioxidant and immunomodulatory effects of ozonated saline solution (O_3_SS) on murine microglial BV2 cells and human endothelial HUVEC cells [1]. The study investigates the biological effects of ozonated saline solution (O_3_SS) on murine BV2 microglial and human HUVEC endothelial cells. O_3_ was dissolved in saline at 1, 5, and 10 µg/NmL and applied to assess cytotoxicity, antioxidant activation, and inflammatory modulation. Low doses enhanced proliferation and upregulated Nrf2 and SOD1, indicating antioxidant stimulation, while 10 µg/NmL reduced viability. O_3_SS decreased ROS production in LPS-stimulated microglia and shifted polarization toward an anti-inflammatory phenotype by lowering iNOS and IL-1β and increasing Arg-1 and IL-10 [1]. The findings suggest that low-dose O_3_SS promotes antioxidant and anti-inflammatory responses, supporting potential therapeutic applications.

In our opinion, despite its orderly layout and apparent rigor in describing experimental protocols, the study suffers from substantial methodological flaws, interpretative overreach, and conceptual weaknesses that collectively compromise and raise concerns about the reliability and broader applicability of its conclusions.

These limitations include a problematic rationale, inadequate experimental controls, unjustified extrapolations to systemic therapy, and a central methodological bias concerning the physicochemical behavior of ozone in small-volume in vitro systems compared to its behavior in bulk saline volumes intended for clinical infusion.

## 2. Methodological Considerations

The study’s fundamental bias begins with the assumption that ozone dissolved in saline within microwells of cell cultures behaves identically to ozone dissolved in larger physiological volumes, such as 250 or 500 mL saline solutions used for intravenous infusions. This equivalence is difficult to support based on established physicochemical principles.

Ozone is a highly unstable gas with a half-life of seconds in aqueous media; its reactivity is profoundly influenced by the diffusion path, temperature, ionic strength, gas–liquid interface area, and the ratio of gas to liquid. In the confined geometry of a microwell, ozone dissolution, diffusion, and reaction kinetics are fundamentally different from those in a macroscopic volume where stratification, incomplete mixing, and rapid decay generate heterogeneous microenvironments. Moreover, the total ozone dose per cell in vitro is drastically higher than that which tissues or plasma components would encounter in vivo, where antioxidant systems, proteins, and lipids immediately quench reactive species.

The assumption that the redox events observed in a microplate correspond to those occurring in clinical O_3_SS infusion represents a significant bias in the rationale, making the extrapolation of results from microwells to systemic application misleading. The authors appear unaware that the ozone concentration declared (1–10 µg/NmL) in microvolumes corresponds to radically different oxidative potentials and exposure kinetics than in a liter-scale solution, where the concentration gradient, gas–liquid equilibrium, and subsequent redox chemistry are vastly altered. This conceptual oversight undermines the translational value of the entire study [1].

Another critical weakness is the reliance on immortalized BV2 microglial cells as a proxy for primary microglia [1]. BV2 cells exhibit significant transcriptomic divergence from primary microglia, including differences in cytokine response, receptor expression, and oxidative stress sensitivity [2]. The extrapolation of results from BV2 to primary microglia or to in vivo neuroinflammatory processes is therefore precarious. Similarly, the use of HUVECs to evaluate potential systemic cytotoxicity is overly simplistic; these cells, isolated from umbilical veins, do not represent the endothelium of adult human vasculature or replicate the dynamic flow and immune interactions of living tissue [3]. The combination of these two cell types cannot model systemic or neuroimmune effects, yet the discussion extends the findings toward therapeutic implications for neurodegenerative and vascular diseases.

Methodologically, the experimental design is narrow and lacks proper controls [1]. The study limits its ozone exposure to three nominal concentrations without confirming the actual ozone content in the cell medium after mixing or during incubation. The assumption that the real dissolved ozone concentration corresponds to 10% of the generator output is based on previous literature rather than direct chemical quantification in the present study. Given the extreme reactivity and short lifetime of ozone in saline, the absence of precise, contemporaneous measurement of dissolved ozone represents a serious flaw. Additionally, the authors’ use of a colorimetric assay to exclude hypochlorite formation as a confounder lacks validation; no independent calibration curve or sensitivity data are provided, and the conclusion that hypochlorite formation was “negligible” rests on the detection limit of the assay, not on actual absence [1]. This form of negative evidence does not demonstrate the chemical purity of the test medium.

Despite the many efforts to demonstrate the reliability of their rationale, the authors interpret changes in mRNA expression as evidence of biological modulation without verifying whether transcriptional upregulation translates to functional protein expression or measurable biochemical outcomes [1]. For example, increases in Nrf2 and SOD1 transcripts are treated as confirmation of antioxidant activation, but there is no measurement of enzymatic activity, redox balance, or total antioxidant capacity [1]. Similarly, the interpretation of decreased IL-1β and increased IL-10 mRNA as a definitive shift toward an “anti-inflammatory phenotype” is simplistic. Microglial polarization is a continuum involving multiple pathways beyond Arg-1 and IL-10; reducing the complexity of inflammatory signaling to a binary M1/M2 dichotomy reflects an outdated conceptual model [4]. Without proteomic confirmation, cytokine secretion assays, or functional endpoints (such as phagocytic capacity or neuroprotective activity), the study’s biological conclusions remain speculative.

Concerns have to be highlighted also for statistics.

The authors’ statistical analysis appears superficial and potentially misleading [1]. They repeatedly report *p*-values of high significance (e.g., *p* < 0.0001) in experiments with only three biological replicates per condition. Such extreme statistical precision is improbable in biological systems with small sample sizes and high inherent variability. Moreover, there is no correction for multiple comparisons despite numerous pairwise tests. This inflates the likelihood of false positives, casting doubt on the reliability of the reported differences.

A further confounding issue concerns the absence of proper negative and positive controls [1]. There is no inclusion of known anti-inflammatory or antioxidant compounds (e.g., dexamethasone or N-acetylcysteine) for benchmarking. Without comparative agents, the relative efficacy of ozone is indeterminate. Likewise, the saline-only control, though mentioned, is insufficient to distinguish effects caused by ozone reaction by-products, such as ozonides or lipid oxidation products, from effects of ozone itself. The authors assume that because hypochlorite was not detected, all observed effects must derive from ozone directly, overlooking the complex chemistry of ozonation, which generates a cascade of secondary reactive oxygen species and aldehydic intermediates that can influence cellular behavior independently of ozone.

## 3. Interpretations of Data

The discussion section appears to emphasize supportive interpretations and citations, potentially limiting the scope of alternative viewpoints.

The authors almost exclusively reference studies supportive of ozone therapy, many of which originate from low-impact journals or self-cited works within the pro-ozone research community [1]. They ignore extensive literature documenting the toxicity, instability, and unpredictable reactivity of ozone in biological fluids. Claims that O_3_SS administration is “safe and well tolerated” rely on anecdotal or non-randomized clinical observations, not rigorous controlled trials. The text consistently frames ozone as a benign “eustress inducer,” adopting promotional language more typical of advocacy literature than critical scientific discourse. Statements such as “O_3_SS may represent a promising adjunctive approach” are not supported by mechanistic evidence or pharmacokinetic understanding of how ozone-derived species interact within the bloodstream, where immediate quenching by antioxidants and proteins would prevent the oxidative cascades hypothesized by the authors.

An additional weakness lies in the absence of temporal kinetics and dose–response modeling. The study measures responses at only two time points, 4 and 24 h, without intermediate assessments or replication of exposure to mimic repeated therapeutic sessions [1]. Ozone effects are known to be transient, often triggering compensatory antioxidant mechanisms that can reverse over time. Without kinetic data, the study cannot claim that the observed transcriptional changes represent stable or beneficial modulation rather than acute stress responses.

The authors’ claim that the study provides insight into systemic therapy mechanisms is therefore unjustified [1]. Ozone in vitro interacts directly with cells in an environment devoid of the plasma antioxidants, proteins, and lipids that dominate the chemistry of ozone in vivo. In actual O_3_SS infusion, ozone reacts almost instantaneously with chloride, bicarbonate, and organic solutes, producing peroxides and radicals that have fleeting existence. The chemical identity of what reaches tissues after an O_3_SS infusion is fundamentally different from what is present in microwells immediately after ozone exposure. Consequently, the entire premise that in vitro findings mirror systemic effects is misleading. The lack of discussion of ozone decay kinetics, solubility limits, and heterogeneous reactivity constitutes a major conceptual flaw.

The discussion reads less like a critical interpretation of data and more like a justification of an already held belief in ozone benefits. There is little recognition of alternative explanations or limitations beyond a perfunctory paragraph at the end that minimizes the in vitro constraint.

In conclusion, the study’s apparent rigor masks significant weaknesses. Its rationale conflates microscale in vitro reactions with macroscopic clinical infusions, a conceptual error that invalidates translational extrapolation. Methodological shortcuts, inadequate controls, overinterpretation of gene expression data, and pervasive selective emphasis on supportive literature render its conclusions unreliable. Without precise chemical characterization of the ozonated medium, validation of biological endpoints at the protein or functional level, and realistic modeling of in vivo exposure conditions, the work cannot substantiate claims that ozone saline solution promotes an anti-inflammatory phenotype or constitutes a safe systemic therapy. The assumption that ozone dissolved in microwells behaves like ozone in a 500 mL physiological saline bag is a critical bias that invalidates the study foundation, as the physicochemical environment, reaction kinetics, and oxidative exposure are incomparable. The result is a paper that, while polished in form, is methodologically limited in its translational applicability, offering more of a reiteration of ozone therapy advocacy than a robust experimental contribution to biomedical science.

## 4. Physical–Chemical Evaluation

Using Ordinary Differential equations (ODEs) in modeling the experimental setting described by Armeli et al. [1]:

(1) $$\frac{dC}{dt}=-kC\;\text{where}\;C\left(t\right)=C_{0}e^{-kt}$$

We showed the following: (a) the volume of dilution drastically reduces *C*_0_, the initial concentration; (b) the decay rate *k* (assumed 0.15 min^−1^) ensures that even small initial concentrations decay fast; (c) in larger volumes, even high total doses cannot overcome the loss of concentration due to dilution.

**Figure 1:**
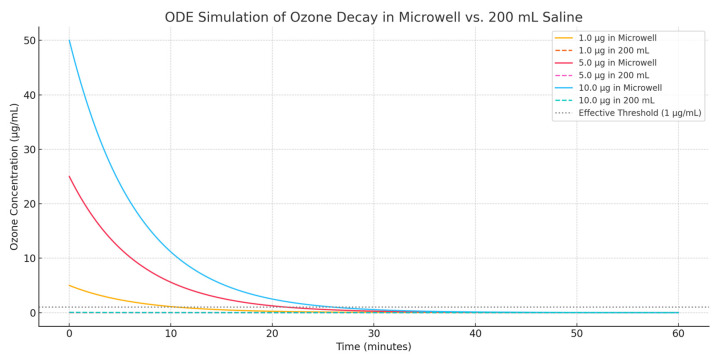
This figure shows ozone concentration decay over 60 min in a microwell and 200 mL saline, using ODEs. Higher doses yield therapeutic levels only in microwells. All 200 mL cases remain below the 1 µg/mL effectiveness threshold throughout the period.

This ODE-based simulation shows how ozone concentration decays over time: (a) microwell (0.2 mL); (b) 200 mL physiological saline for three different doses: 1 µg, 5 µg, and 10 µg (Figure 1).

We emphasize that this modeling approach is intended to evaluate the plausibility of achieving therapeutic ozone concentrations in clinical settings. It does not contradict the in vitro findings, which are valid within their specific experimental context.

The basic interpretation is that in the microwell, even the 1 µg dose starts at 5 µg/mL, above the effective biological range (1–5 µg/mL) and all doses remain effective (>1 µg/mL) for a significant portion of the 60 min. In 200 mL saline, all doses start far below the therapeutic range: (a) 1 µg → 0.005 µg/mL, (b) 5 µg → 0.025 µg/mL, and (c) 10 µg → 0.05 µg/mL. Furthermore, the concentration never reaches the 1 µg/mL threshold, even at the highest dose. Thus, ozone’s anti-inflammatory action is effectively lost when diluted into 200 mL of 0.9% NaCl.

Besides dilution, concerns should be raised about the different physicochemical microenvironment in microwells with respect to the bulk volume of the saline solution in an infusion bottle.

We model ozone concentration C(t) over time using the modified ODE:

(2) $$\frac{dC}{dt}=-k_{decay}C-k_{bio}C$$

where k_decay_ is the physicochemical decomposition rate, temperature and pressure-dependent and k_bio_ biological consumption rate (important in microwell with cells). We model ozone loss as a sum of (a) spontaneous chemical decay; (b) consumption by reactive cellular components.

From aqueous ozone decay kinetics (literature + empirical):

(3) $$k_{decay}\left(T\right)\approx \;k_{0}\cdot e^{\beta \left(T-T_{0}\right)}$$

Assuming the following: (a) T_0_=298 K (25 °C); (b) k_0_ = 0.15 min^−1^ at 25 °C; (c) β ≈ 0.07 per °C (based on Arrhenius-like behavior). Furthermore, the biological decay k_bio_ can be reported as follows: (a) only relevant in microwells, where cells actively consume ozone via redox interactions. (b) estimate: k_bio_∼0.1–0.3 min^−1^. Calculating these ODEs k_bio_ in microwells can correspond to 0.2 min^−1^ (with cells), whereas k_bio_ = 0 in 200 mL saline solution (without cells), and k_decay_ is 0.15·e^(0.07·(T−298))^ and 0.15 min^−1^, respectively.

Ozone lifespan results (ODE-Based) captured from the physical–chemical simulation, consider that for microwell (37 °C, with cells) and lifespan ≈ 8.4 min, with a rapid decay due to both thermal instability and biological consumption; whereas in 200 mL saline (25 °C, no cells) and lifespan ≈ 30.9 min, where it could be forecast a slower decay due to lower temperature and absence of reactive cellular demand.

This should occur only in a theoretical, ideal way.

Actually, ozone is not at all stable in the bulk saline solution.

While our prior model showed faster depletion in microwells due to cell-mediated ozone scavenging, in real aqueous systems, such as saline solutions, ozone is unstable and rapidly decomposes into secondary species, such as hydrogen peroxide (H_2_O_2_) and radicals (•OH, O_2_^−^). This non-biological decomposition is accelerated by the following: (a) trace metals (e.g., Fe^2+^ and Cu^2+^); (b) halide ions (Cl^−^ in NaCl); (c) high gas–liquid interface (e.g., in a bottle headspace). Absence of ozone-consuming cells means no antioxidant buffering: ozone reacts aggressively with solvent or solutes.

In the literature, ozone half-life in 0.9% NaCl at room temperature (no cells) has a t_1_/_2_ ≈ 2–3 min, even without biological materials [5]. Whereas, in microwells, ozone reacts immediately with lipids, thiols, and antioxidants, but those reactions may transiently preserve oxidant signaling species (e.g., 4-HNE, H_2_O_2_). So, paradoxically, ozone decays faster in saline bags (0.7 min^−1^) than in microwells with cells (0.4 min^−1^), as updated *k* values.

Contrary to intuition, ozone persists longer in microwell culture systems (11.6 min) than in saline bags (6.6 min), where it quickly transforms into hydrogen peroxide and other oxidants. This confirms your concern and aligns with both theoretical chemistry and experimental kinetics.

In this perspective, we wondered how much lipoperoxides, particularly 4-HNE, are formed if using (a) a microenvironment enriched with cells (for example, the whole blood in a major oxygen–ozone autohaemotherapy O_2_-O_3_-MAHT), and (b) a microenvironment lacking cells (for example, saline solution in a bag for systemic infusion).

We can describe a (a) fast infusion, as 8 mg ozone is mixed rapidly (as gas) in 200 mL blood at 37 °C immediately generating 4-HNE; (b) slow infusion, as 8 mg ozone is dissolved into 200 mL saline, then infused slowly over 30 min into blood and there producing 4-HNE.

Ozone reacts rapidly and quantitatively in blood with unsaturated lipids, yielding 4-HNE as a major product from peroxidation of ω-6 polyunsaturated fatty acids (mainly linoleic acid). The literature suggests one mole of ozone yields ≈ 0.5 mole of 4-HNE (reaction efficiency ~50%) [6]. In saline, ozone forms mainly H_2_O_2_, not 4-HNE, because it lacks lipids.

So, in scenario (a): a large amount of ozone is bioavailable for lipid peroxidation → high 4-HNE; in scenario (b): ozone is lost during slow infusion → minimal 4-HNE generated.

In converting ozone mass to moles: molar mass of O_3_ = 48 g/mol, so:

$$n_{O_{3}}=\frac{8\;mg}{48\;g/mol}=\frac{0.008}{48}\approx 1.67\cdot 10^{-4}mol$$

To estimate the 4-HNE yield, using 50% molar conversion [6]:

$$n_{4-HNE}=0.5\cdot nO_{3}=0.5\cdot 1.67\cdot 10^{-4}=8.33\cdot 10^{-5}mol$$

In 200 mL of blood: $C_{4-HNE}=\frac{8.33\cdot 10^{-5}mol}{0.2\;L}=4.17\cdot 10^{-4}mol/L\;=\;417\;\mu mol/L$

From our previous simulations, ozone decays significantly in saline water, with only ~23% remaining after 30 min (from ODEs).

So:

$$n_{O_{3effective}}\approx 0.23\cdot 1.67\cdot 10^{-4}\approx 3.84\cdot 10^{-5}mol$$

$$n_{4-HNE}\approx 0.5\cdot 3.84\cdot 10^{-5}=1.92\cdot 10^{-5}mol$$

$$C_{4-HNE}=\frac{1.92\cdot 10^{-5}}{0.2}=9.6\cdot 10^{-5}mol/L=96\;\mu mol/L$$

Fast, direct exposure to ozone in blood leads to ~4.3× more 4-HNE, confirming that route and speed of delivery drastically alter redox metabolite output.

## 5. Limitations and Further Considerations

### 5.1. About the Study

We acknowledge that the original study was conducted under in vitro conditions and that the authors did include a “Limitations” section acknowledging the constrained scope of their experimental model and the necessity for further in vivo and pharmacokinetic studies. We fully agree that such transparency is commendable and essential for scientific integrity.

However, our commentary aims to go beyond a general acknowledgment of model limitations by addressing several *specific interpretative claims and translational inferences* that, in our assessment, extend beyond what the experimental data can support—even within the bounds of a preliminary study. Below, we highlight the most significant examples where we believe the conclusions or implications presented in the original article may not be adequately supported, even in light of the authors’ stated limitations:

1. Therapeutic Framing in Abstract and Discussion:
  Despite noting limitations, the article refers to ozonated saline as a potential “adjunctive approach” and implies that the in vitro observations may underlie systemic therapeutic effects. For instance, the conclusion that “O_3_SS may represent a promising adjunctive approach in inflammatory or neurodegenerative conditions” goes beyond data derived solely from BV2 and HUVEC cells and lacks corresponding pharmacokinetic modeling or in vivo corroboration.
2. Unjustified Translational Extrapolation:
  The assumption that effects observed in microwell exposures (at µg/mL concentrations) could be replicated in clinical infusion volumes ignores key physical–chemical constraints. While the authors note the need for in vivo studies, they do not clearly delineate the magnitude of the disparity between microenvironmental ozone behavior and clinical settings. Our kinetic simulations and decay modeling specifically illustrate how the proposed translational trajectory is chemically implausible under real-world infusion conditions.
3. Absence of Direct Quantification:
  While limitations of in vitro models are mentioned, the lack of direct ozone concentration measurements—despite relying on specific dosimetry for biological interpretation—remains unaddressed. This omission is critical, as it undermines the validity of dose–response interpretations.
4. Functional Overreach Based on Gene Expression:
  Conclusions about antioxidant or anti-inflammatory phenotypes are primarily based on mRNA expression changes, without protein-level validation or functional assays. While the limitations section acknowledges the in vitro nature of the model, it does not explicitly question the strength of causal interpretation or the lack of confirmation at the protein or signaling level.
5. Statistical Inference Precision:
  The article reports highly significant *p*-values (e.g., *p* < 0.0001) from experiments with only three biological replicates per group. Although a small sample size is acknowledged, the potential inflation of significance and the lack of multiple comparison correction are not critically examined in the original limitations section.

In summary, we agree that the original authors demonstrated a degree of caution in their interpretation, but we believe our commentary provides additional specificity on why certain conclusions—especially those implying systemic therapeutic potential—are not just premature, but physically and biochemically implausible based on the data and model used. By delineating these points, we aim to sharpen the boundaries between acceptable preliminary observation and unsupported extrapolation.

### 5.2. Simulation with ODEs

We would like to clarify that our ODE-based modeling was never intended to invalidate the in vitro findings themselves. The cellular responses observed under controlled experimental conditions—such as modulation of redox-sensitive genes—are acknowledged as valid within that specific microenvironment. Our critique is directed not at the in vitro data per se, but at the translational interpretation of those results as indicative of systemic therapeutic potential.

The purpose of the kinetic modeling was to provide a quantitative framework to evaluate whether similar ozone concentrations—and therefore similar biological exposures—could plausibly be achieved in clinically relevant settings, such as intravenous infusion using ozonated saline. By simulating ozone decay under different volumes, temperatures, and biological contexts, our goal was to show that the physicochemical behavior of ozone in bulk saline diverges significantly from that in microwell cultures. These simulations highlight a mechanistic barrier to extrapolation, not an invalidation of the original in vitro observations.

## 6. Conclusions

The findings and critical analysis presented in this work lead to a necessary re-evaluation of the assumptions, methodologies, and translational claims surrounding the use of ozonated saline solutions in biomedical contexts. While the study by Armeli et al. aims to highlight the potential antioxidant and immunomodulatory effects of low-dose ozone exposure via physiological saline, a more careful and mechanistically grounded interpretation raises doubts about both the validity of the experimental model and the generalizability of the results.

Ozone is an extremely reactive molecule whose behavior in biological systems is strongly dependent on the physical and chemical context in which it is introduced. In microplate assays, where small volumes of solution are in direct and immediate contact with living cells, ozone rapidly interacts with cellular membranes, thiol groups, and lipids, generating reactive intermediates such as hydrogen peroxide and aldehydic lipid peroxidation products like 4-hydroxynonenal. In this environment, the brief presence of ozone or its byproducts can indeed initiate biological responses, including activation of Nrf2, upregulation of antioxidant enzymes, and modulation of inflammatory gene expression. However, the concentration of ozone in these microwell systems is not representative of clinical ozone therapy.

The absolute amount of ozone used may be low, but the effective concentration—measured in micrograms per milliliter—can be orders of magnitude higher than what is achieved when the same dose is diluted in clinically relevant volumes of saline.

When ozone is instead introduced into a large volume of physiological saline, as would occur during intravenous infusion protocols, its chemical reactivity leads to rapid decomposition. It reacts with chloride ions, dissolved oxygen, water, and other minor constituents of the solution, resulting primarily in the formation of oxygen gas and small amounts of hydrogen peroxide. Without the presence of lipids or reactive biomolecules, the ozone cannot produce lipid-derived mediators, and the generation of signaling molecules like 4-HNE is effectively negligible. Furthermore, slow infusion exacerbates this problem by allowing more time for spontaneous decomposition before any biological interaction can occur. Kinetic modeling and mass balance analysis suggest that, under plausible clinical conditions, the majority of ozone may decay before reaching systemic circulation. These theoretical considerations are offered to inform the feasibility of translational application, not to challenge the biological effects observed in vitro.

The commentary also identifies key limitations in the biological models used. While BV2 and HUVEC cells provide a convenient and reproducible platform for in vitro screening, they do not faithfully replicate the complexity of immune and endothelial systems in vivo. Immortalized cell lines often lack the regulatory networks and epigenetic landscapes of primary cells, leading to exaggerated or non-physiological responses. Moreover, reliance on transcriptional data without corresponding protein or functional assays limits the interpretability of the findings. Gene expression alone cannot be equated with biological effect, particularly in the context of oxidative signaling, which is highly dynamic and temporally constrained.

In sum, while the observed gene expression changes in microcultures exposed to ozonated saline are interesting from a redox biology standpoint, they do not support the claim that ozone dissolved in saline is a therapeutically effective agent when administered systemically. The physicochemical properties of ozone, including its short half-life and high reactivity, mean that its effects are highly dependent on the medium of delivery, contact time, and presence of reactive substrates. In blood, where these conditions are favorable, ozone is rapidly converted into bioactive products that can trigger controlled oxidative signaling. In saline, these conversions do not occur to any appreciable degree. Thus, the route of administration and the context in which ozone is delivered are more important than the dose alone. Any future therapeutic use of ozonated fluids must take into account these critical biochemical realities.

## Data Availability

Data are available on request to the Corresponding Author (S.C.).

## Abbreviations

O_3_SS—Ozonated Saline Solution; BV2—A murine (mouse-derived) immortalized microglial cell line; HUVEC—Human Umbilical Vein Endothelial Cells; O_3_—Ozone; Nrf2—Nuclear Factor Erythroid 2–Related Factor 2; SOD1—Superoxide Dismutase 1; ROS—Reactive Oxygen Species; LPS—Lipopolysaccharide; iNOS—Inducible Nitric Oxide Synthase; IL-1β—Interleukin-1 beta; Arg-1—Arginase-1; IL-10—Interleukin-10; ODEs—Ordinary Differential; Equations; NaCl—Sodium Chloride; 4-HNE—4-Hydroxynonenal; O_2_-O_3_-MAHT—Major Autohemotherapy with Oxygen–Ozone; µg/NmL—Micrograms per Normal Milliliter (though “NmL” seems non-standard; possibly an error for µg/mL); mRNA—Messenger Ribonucleic Acid.
